# The Relationship Between Demographic, Socioeconomic, and Health-Related Parameters and the Impact of COVID-19 on 24 Regions in India: Exploratory Cross-Sectional Study

**DOI:** 10.2196/23083

**Published:** 2020-11-27

**Authors:** Ravi Philip Rajkumar

**Affiliations:** 1 Jawaharlal Institute of Postgraduate Medical Education and Research Pondicherry India

**Keywords:** burden of disease, COVID-19, diarrheal disease, ischemic heart disease, population size, sex ratio

## Abstract

**Background:**

The impact of the COVID-19 pandemic has varied widely across nations and even in different regions of the same nation. Some of this variability may be due to the interplay of pre-existing demographic, socioeconomic, and health-related factors in a given population.

**Objective:**

The aim of this study was to examine the statistical associations between the statewise prevalence, mortality rate, and case fatality rate of COVID-19 in 24 regions in India (23 states and Delhi), as well as key demographic, socioeconomic, and health-related indices.

**Methods:**

Data on disease prevalence, crude mortality, and case fatality were obtained from statistics provided by the Government of India for 24 regions, as of June 30, 2020. The relationship between these parameters and the demographic, socioeconomic, and health-related indices of the regions under study was examined using both bivariate and multivariate analyses.

**Results:**

COVID-19 prevalence was negatively associated with male-to-female sex ratio (defined as the number of females per 1000 male population) and positively associated with the presence of an international airport in a particular state. The crude mortality rate for COVID-19 was negatively associated with sex ratio and the statewise burden of diarrheal disease, and positively associated with the statewise burden of ischemic heart disease. Multivariate analyses demonstrated that the COVID-19 crude mortality rate was significantly and negatively associated with sex ratio.

**Conclusions:**

These results suggest that the transmission and impact of COVID-19 in a given population may be influenced by a number of variables, with demographic factors showing the most consistent association.

## Introduction

The COVID-19 pandemic, caused by the novel coronavirus SARS-CoV-2, has emerged as perhaps the most significant health crisis of our time [1]. An unexpected observation in the context of this pandemic has been the wide variations in prevalence, mortality rate, and case fatality rate across affected countries, which cannot be wholly explained on the basis of differences in the virulence of SARS-CoV-2 strains [2-5]. While some of this variation may reflect differences in health care and testing capacity across nations, it remains important to examine the role of other factors in causing this variability, particularly socioeconomic determinants of health [6,7]. There is already evidence that social factors, such as perceived sociability, socioeconomic disadvantage, health literacy, trust in regulatory authorities, and the speed and stringency of measures instituted to control the spread of COVID-19, can crucially influence these variables [1,2,7]. These social factors interact with individual psychological responses to influence behavior either positively or negatively—for example, an adaptive (“functional”) level of fear of COVID-19 was associated with better adherence to public health safety measures in an international sample of adults, while self-reported depression had the opposite effect [8]. Preliminary research has found that demographic and socioeconomic factors can influence variability in the spread and impact of COVID-19 not only between countries but within a given country; in an ecological analysis of data from the United States, poverty, number of elderly people, and population density were positively correlated with COVID-19 incidence and mortality rates [9].

At the time of writing this paper, India ranked third among all nations in terms of the total number of confirmed cases of COVID-19, following the United States and Brazil, with over 3,000,000 cases reported as of August 25, 2020 [10]. Following the initial identification of 563 positive cases, the Indian government instituted a nation-wide lockdown for a period of 21 days, which began at midnight on March 24, 2020, and was gradually relaxed over the next 2 months [11]. Data from the initial phase of the lockdown suggested that this measure significantly reduced the transmission of COVID-19; however, this number rapidly increased in subsequent months. This rapid increase was not uniform: across the 32 states and territories of India, certain states have reported over 1000 cases, while others have reported far lower numbers despite their geographical proximity to these states [12,13].

Besides the demographic and socioeconomic variables discussed above, an important factor that may influence such variations in the Indian context is the availability and quality of health care. Health care facilities in India are unevenly distributed, with a significant urban-rural divide, and this inequality has been further exacerbated by the COVID-19 pandemic [14,15].

Keeping the above in mind, an exploratory study was conducted to examine the relationship between demographic, socioeconomic, and health-related indices and measures of the spread and impact of COVID-19 across different states in India. These indices were drawn both from published research to date and from factors hypothesized to influence the spread and outcome of COVID-19.

## Methods

### COVID-19–Related Data

The current study was an exploratory cross-sectional study based on data officially released by the Government of India. Information related to COVID-19 was obtained from the website of the Ministry of Health and Family Welfare, which provides information on the total number of cases, active cases, recovered cases, and deaths for each state and territory of India and is updated every 24 hours [16]. Data for this study were recorded from the above source on June 30, 2020. Out of the 32 states and territories, only the 24 regions that reported at least 500 cases and one or more deaths were selected, to permit a meaningful computation of COVID-19–related indices.

After obtaining information on the population of each region from the Government of India’s official census data [17], and verifying it against updated projections for 2020 from the Unique Identification Authority of India [18], the following indices related to COVID-19 were calculated for each state:

- The estimated prevalence rate: the total number of cases (active, recovered, and deceased) per 1 million population
- The crude mortality rate: the total number of reported deaths due to COVID-19 per 1 million population
- The case fatality rate: the ratio of deaths to all cases with outcomes (death or recovery), expressed as a percentage.

### Demographic Information

Details on population per state were recorded using the census data cited above, as well as the updated population projections for the year 2020 provided by the Unique Identification Authority of India, while information on population density was obtained from the National Institution for Transforming India (NITI-Aayog), the Government of India’s official source of data on demographic and socioeconomic variables [17-19]. As age and male sex have both been associated with mortality due to COVID-19, mean life expectancy for each state and male-to-female sex ratio per state, defined as the number of females per 1000 male population, were obtained from the same source [9,20].

### Socioeconomic Variables

Information on literacy rates and female literacy rates per state was obtained from official census data, while information on poverty, defined as the percentage of people living below the poverty line in each state, was obtained from the data published by the Ministry of Social Justice and Empowerment [21]. Information on indices related to law and order—statewise rates of homicide, accident, and rape—were obtained from the official statistics published in 2018 by the National Crime Records Bureau [22]. This information was included due to the proposed role of law enforcement, and adherence to it, in containing the spread of COVID-19 [2]. As international air travel has also been linked to the spread of COVID-19, information on which states had a functional international airport was obtained from the website of the Airports Authority of India [23,24].

### Health-Related Variables

Information on a number of general indices of health for each state—the maternal, infant, and under-five mortality rates and the percentage of children under 24 months who were fully immunized—was obtained from official NITI-Aayog data, which was updated for the 2015-2016 fiscal year. In addition, information on the percentage of disability-adjusted life years (DALYs) for 6 common health conditions—diarrheal disease, lower respiratory infection, tuberculosis, diabetes mellitus, chronic obstructive pulmonary disease, and ischemic heart disease—was obtained from the official report on state-level disease burden commissioned by the Department of Health Research, Ministry of Health and Family Welfare, and published in 2017 [25]. These variables were studied due to the emerging evidence on the role of medical comorbidities in determining the outcome of COVID-19, as well as the hypothesized role of past infectious diseases in influencing the host immune response to SARS-CoV-2 [3,26]. In view of the proposed relationship between depression and decreased adherence to public health measures, estimated statewise prevalence rates of depression were obtained from the 2017 Global Burden of Disease Study [27]. A complete list of the data sources used in this study is provided in Table 1.

**Table 1:** Official data sources used for the analyses in this study.

Study variable	Data source
Total population per state	Census of India [17]; Unique Identification Authority of India projections [18]
Population density per state; mortality indices per state	National Institution for Transforming India (NITI Aayog) [19]
Literacy rates per state	Census of India [17]
Percentage living below the poverty line per state	Ministry of Social Justice and Empowerment [21]
Crime-related statistics per state	National Crime Records Bureau Report, 2018 [22]
Presence of international airports in a given state	Airports Authority of India [24]
Disease burden per state	India: Health of the Nation’s States – the India State-Level Disease Burden Initiative [25]
Estimated prevalence of depression per state	Global Burden of Disease Study, Indian data [27]
COVID-19 statistics	Ministry of Health and Family Welfare [16]

### Ethical Issues

This study was based on an analysis of data available in the public domain and did not involve any human subjects. As per the Institute Ethics Committee guidelines of the author’s institution, such analyses do not require formal approval by the committee.

### Data Analysis

Data were analyzed using SPSS, version 20.0 (IBM Corp). Prior to bivariate analysis, all study parameters were tested for normality. As the COVID-19 indices—prevalence, mortality rate, and case fatality rate—were not normally distributed (*P*<.01 for all indices, Shapiro-Wilk test), the Spearman rank correlation coefficient (ρ) was used to test the hypothesis of a monotonic relationship between these indices and the aforementioned demographic, socioeconomic, and health-related indices. For the purpose of this study, a significance level of *P*<.05 was considered significant. This value carries with it a certain risk of maximizing the significance of marginal or potentially false-positive findings; however, given the exploratory nature of this study, it was adopted in order to avoid rejecting potentially significant associations on the basis of a more or less arbitrary cut-off value [28,29].

To confirm the strength of these associations, a multivariate linear regression was carried out for each of the individual COVID-19 indices. Only those variables that were associated with these indices at a significance of *P*<.05 or below in univariate or multivariate analyses were included in the multivariate analyses for each index.

### Data Availability Statement

All data used in this study were obtained from public-domain data sources (Table 1). A complete data set is available in Multimedia Appendix 1.

## Results

### Sample Description

Data were obtained for 23 Indian states and one territory (Delhi) for the period up to June 30, 2020. As of this date, 566,840 confirmed cases of COVID-19, and 17,337 deaths due to the disease, had been officially reported. The mean and standard deviation values of prevalence, crude mortality rate, and case fatality rate for the entire sample were 504.13 (SD 896.64) cases per 1 million population, 12.68 (SD 30.03) deaths per 1 million population, and 2.77 (SD 2.21) deaths per 100 cases. respectively. There was a wide range of variation across the COVID-19 indices, with prevalence ranging from 61.25 (Jharkand) to 4440.03 (Delhi) per 1 million population, mortality ranging from 0.24 (Tripura) to 140.19 (Delhi) per 1 million population, and case fatality rate ranging from 0.09% (Tripura) to 7.9% (Maharashtra) (see Multimedia Appendix 2 for the complete details.)

Correlations between the demographic, socioeconomic, and health-related indices listed above and the COVID-19 indices are provided in Table 2. The raw data underlying all these analyses are available in Multimedia Appendix 1.

**Table 2:** Relationship (Spearman rank correlation coefficient [ρ]) between demographic, socioeconomic, health-related, and COVID-19 indices in 24 Indian regions for the period ending June 30, 2020. Values significant at *P*<.05 are indicated in italics.

Variable			COVID-19 estimated prevalence		COVID-19 crude mortality rate		COVID-19 case fatality ratio	
			ρ	*P* value	ρ	*P* value	ρ	*P* value
**Demographic indices**								
	Total population		–0.240	.26	0.225	.29	0.526^*^	.009^*^
	Population density		–0.129	.57	–0.051	.82	0.092	.68
	Sex ratio		–0.571^*^	.008^*^	–0.517^*^	.02^*^	–0.369	.10
**Indices of human development**								
	Life expectancy		0.469^*^	.03^*^	0.489^*^	.02^*^	0.230	.32
	Percentage living below the poverty line		–0.466^*^	.04^*^	–0.418	.06	–0.251	.27
	Literacy rate		0.453^*^	.03^*^	0.155	.48	–0.030	.89
**Indices of law and order**								
	Homicide rate		–0.171	.43	–0.313	.14	–0.364	.08
	Rape rate		–0.044	.84	–0.052	.81	–0.149	.49
	Accidental death rate		–0.350	.10	–0.309	.15	–0.149	.50
**Health indicators**								
	Maternal mortality rate		0.353	.15	–0.499^*^	.04^*^	–0.330	.18
	Infant mortality rate		–0.474^*^	.02^*^	–0.276	.19	–0.090	.68
	Under-five mortality rate		–0.406	.08	–0.476^*^	.04^*^	–0.304	.21
	**Disability-adjusted life years**							
		Diarrheal disease	–0.563^*^	.004^*^	–0.500^*^	.01^*^	–0.259	.22
		Tuberculosis	–0.224	.29	0.024	.91	0.121	.57
		Chronic obstructive pulmonary disease	0.225	.29	0.262	.22	0.295	.16
		Lower respiratory tract infection	–0.338	.12	–0.256	.23	–0.135	.53
		Diabetes mellitus	0.348	.16	0.244	.33	0.173	.49
		Ischemic heart disease	0.476^*^	.02^*^	0.535^*^	.007^*^	0.367	.08
		Iron deficiency anemia	–0.116	.60	0.030	.89	0.125	.57
	Depression, estimated prevalence		0.373	.09	0.176	.43	0.006	.98
	Suicide rate		0.261	.22	0.090	.67	0.020	.93

^*^P values <.05

### Relationship Between Demographic Variables and COVID-19 Indices

COVID-19 prevalence for each region was significantly and negatively correlated with sex ratio (*P*=.008) and was positively associated with life expectancy (*P*=.03) (Table 2). Crude mortality rate was positively correlated with life expectancy and negatively correlated with sex ratio (both *P*<.05). In contrast, the case fatality rate was significantly correlated with the total population of each region (*P*<.009).

### Relationship Between Socioeconomic Variables And COVID-19 Indices

The prevalence of COVID-19 showed a positive association with the life expectancy and literacy rate, and a negative trend-level association with the percentage of people living below the poverty line (all *P*s<.05) (Table 2). No significant correlations were observed between COVID-19 mortality and case fatality rates and any socioeconomic parameter, though a negative association of marginal significance was observed between the percentage of individuals living below the poverty line and the crude mortality rate (*P*=.06). As the presence or absence of an international airport was a dichotomous variable and the COVID-19 indices were not normally distributed, the Mann-Whitney *U* test was used to compare these indices. States with an international airport had a significantly higher estimated COVID-19 prevalence (*U*=118.0, *P*=.007) but did not differ significantly in terms of mortality or case fatality. None of the putative indices of law and order were significantly associated with any COVID-19 parameters.

### Relationship Between Health-Related Variables and COVID-19 Indices

COVID-19 prevalence was significantly and negatively correlated with the burden of diarrheal disease per state (*P*=.004) and the infant mortality rate at *P*<.05, and was positively associated with the burden of ischemic heart disease (*P*=.02) (Table 2). In contrast, the mortality rate showed a significant positive correlation with the burden of ischemic heart disease (*P*=.007), and was negatively associated with the maternal mortality rate, under-five mortality rate, and burden of diarrheal disease (all *P*s<.05). None of the health-related variables were significantly associated with the case fatality rate. The two indices of mental health—statewise suicide rate and estimated prevalence of depression—were not significantly related to any COVID-19 indices, though a marginal positive association with estimated prevalence was found for depression (*P*=.09).

### Multivariate Analyses

All variables that were significantly associated with COVID-19 indices at a significance level of *P*<.05 or lower were selected for multivariate linear regression analyses. For COVID-19 estimated prevalence, these variables were sex ratio, life expectancy, percentage of the population living below the poverty line, literacy rate, infant mortality rate, and DALYs due to diarrheal disease and ischemic heart disease. The final model explained only 8% of the variance in prevalence (adjusted R^2^=0.080), and analysis of variance yielded an *F* value of 1.248 (df=13), with a significance of *P*=.35, suggesting that the null hypothesis should be retained. None of the individual variables were significantly associated with COVID-19 prevalence in this model.

For the COVID-19 crude mortality rate, the variables entered in the model were sex ratio, life expectancy, maternal mortality rate, under-five mortality rate, and DALYs due to diarrheal disease and ischemic heart disease. The final model explained 20.4% of the variance in crude mortality rate (adjusted R^2^= 0.204) and analysis of variance yielded an *F* value of 1.682 (*P*=.22), again suggesting that the null hypothesis should be retained. However, among individual variables, sex ratio remained significantly and negatively associated with this variable (*t*=–2.361, *P*=.04).

As only a single study variable—the population size—was associated with the case fatality ratio, multivariate analyses were not carried out in this case.

## Discussion

### Principal Findings

The results of this preliminary analysis found that certain demographic, socioeconomic, and health-related variables were significantly related to the variability in COVID-19 prevalence, mortality rate, and case fatality rate across 24 regions in India. In particular, COVID-19 prevalence was associated with sex ratio and the burden of diarrheal disease as measured by the percentage of DALYs associated with this disorder, as well as with the presence of an international airport in a given state; COVID-19 mortality was associated with the burden of ischemic heart disease; and COVID-19 case fatality rate was associated with the total population of each region. The results of the multivariate analyses indicated a negative, significant association between sex ratio and COVID-19 prevalence and mortality.

The association between sex ratio and measures of the impact of COVID-19 is in line with existing research findings. Several clinical case series, both from India and other countries, have reported a preponderance of male patients in hospitalized samples, as well as a link between male sex and mortality due to COVID-19 [30-33]. This phenomenon may be partly explained by sex differences in the immune and inflammatory response to SARS-CoV-2 infection [18]. However, in the Indian context, this relationship could also be influenced by traditionally defined gender roles. These are associated with comparatively greater freedom of movement for men, which places them at a higher risk of exposure to infection [34,35]. The association between the presence of an international airport and the statewise prevalence of COVID-19 is also in line with earlier evidence highlighting the role of international air travel in the transmission of SARS-CoV-2 across nations [23].

Similarly, the link between state-wide differences in the burden of ischemic heart disease and mortality due to COVID-19 is supported by clinical research, which has found an association between the presence of ischemic heart disease and the severity of COVID-19 [36,37]. Moreover, ischemic heart disease is commonly associated with other medical conditions, such as systemic hypertension and chronic renal disease, which themselves worsen the outcome of COVID-19, and COVID-19 has been documented to trigger myocardial injury in patients with pre-existing coronary artery disease [38,39]. No such significant association was found in this study for other medical comorbidities, such as diabetes mellitus or chronic obstructive pulmonary disease. However, such comorbidities have been associated with worse COVID-19 outcomes in clinical samples [37,38]; the failure of this study to confirm this association reflects the limitations inherent in an ecological approach.

Though this could not be confirmed by multivariate analysis, population was positively correlated with the case fatality rate across the different regions of India. This association does not appear to be mediated solely by overcrowding, as no significant association was found between population density and case fatality. A possible explanation for this finding is the unequal distribution and accessibility of health care facilities in India, particularly in areas that have a high total population but a relatively low population density, with limited availability of facilities for testing and treatment in nonurbanized regions [40,41]. Such inequalities may lead to delays in obtaining appropriate treatment [42].

The negative association found between the burden of diarrheal disease and the prevalence of COVID-19 across regions is an unexpected finding, as no such association was found for respiratory diseases such as lower respiratory infection (ρ=–0.227, *P*=.26) or pulmonary tuberculosis (ρ=0.024, *P*=.91). While it has been postulated that prior exposure to respiratory coronaviruses may moderate the impact of SARS-CoV-2 infection, no such association has been suggested or demonstrated thus far for gastrointestinal infections [3]. However, in vitro research has shown that intestinal replication may contribute to the progression of SARS-CoV-2 infection; therefore, it is possible that prior intestinal viral infections, which are a common cause of diarrheal disease, could influence this process [43]. Alternately, this association may be related to behavioral factors, such as reduced population mobility in those with pre-existing gastrointestinal disorders minimizing exposure to SARS-CoV-2, or improved adherence to hand hygiene in those who have experienced prior episodes of diarrheal disease. Finally, this may be a false-positive finding arising from the exploratory nature of the analyses conducted. Though the biological mechanism advanced above has some support in theory, it can neither be confirmed nor disproved using the ecological methods of analysis adopted in this study [44,45].

A number of other associations were observed at a trend level. While the direction of these associations was unexpected in some cases—such as a positive association between COVID-19 prevalence and literacy, and a negative association between COVID-19 and levels of poverty and maternal and under-five mortality rates—these findings must be interpreted with caution, owing to their low statistical significance and the large number of potential confounding factors, as well as the possibility of type I error.

### Limitations

The results of this study must be viewed in light of certain limitations. First, demographic, socioeconomic, and health-related data were obtained from official government statistics and populations, which preceded the onset of the COVID-19 outbreak by a period of 3 to 6 years. Therefore, some of this information may not accurately reflect the contemporary situation in the different states of India. Second, this study did not take into account other factors that could influence the spread of COVID-19, such as cultural norms and practices, local variations in climate and temperature, and the efficiency of implementation of quarantine and related measures [1,2]. Third, the data analysis did not take into account the confounding effects of other variables on the bivariate analyses. Fourth, due to logistic and manpower constraints on testing and case finding, the officially reported statistics on COVID-19 may underestimate the true scope of this problem in India [6,13]. Finally, owing to the cross-sectional nature of this study, it was not possible to assess the relationship between the study variables and trends in the spread of COVID-19, such as the rate of increase in the number of cases.

### Conclusions

In conclusion, the results of this study, though limited by the nature of the exploratory analyses and the study design itself, suggest that some of the factors that have been found to influence the outcome of COVID-19 at a clinical level, such as male sex and comorbid ischemic heart disease, also have an impact at the population level. Other unexpected findings, such as the link between population and case fatality and between diarrheal disease burden and COVID-19 prevalence, may represent potential behavioral, socioeconomic, or biological mechanisms that require further elucidation. Though these results may be considered preliminary, they may aid future researchers in studying some of the specific associations found in this study in more depth, and in understanding the advantages and limitations of the approach adopted in this paper. Moreover, despite their limitations, they illustrate the value of ecological analyses in understanding the COVID-19 pandemic, particularly in situations where direct clinical or epidemiological research is not feasible due to safety measures. Ecological analyses can be carried out relatively rapidly and safely in this setting, and the tentative associations observed using this method can be subject to more rigorous analyses in field settings to confirm or refute their validity. Further longitudinal research with more sophisticated statistical modeling and up-to-date data may clarify the role of these and other demographic, socioeconomic, and health-related variables in moderating the impact of COVID-19 within nations, and may inform future strategies to curtail the impact of this pandemic.

## Appendix

Multimedia Appendix 1 Data set comprising all data used in this study.

Multimedia Appendix 2 Variation in COVID-19 indices across 24 regions in India.

## Abbreviations

DALY: disability-adjusted life year
NITI-Aayog: National Institution for Transforming India
