# Supplementary material for: The Relationship Between Demographic, Socioeconomic, and Health-Related Parameters and the Impact of COVID-19 on 24 Regions in India: Exploratory Cross-Sectional Study
Source: JMIR Public Health Surveill. 2020 Nov 27;6(4):e23083. doi: 10.2196/23083 (PMC7717919; doi:10.2196/23083)
Supplement: Multimedia Appendix 2 [file publichealth_v6i4e23083_app2.doc]

**Supplementary Table 1:** Prevalence, crude mortality and case fatality rates for 24 Indian states as of June 30, 2020.

| **State** | **Number of confirmed COVID-19 cases reported** | **Number of deaths due to COVID-19 reported** | **Number of recovered COVID-19 cases** | **COVID-19 prevalence (cases per 1 million population)** | **COVID-19 crude mortality rate (deaths per 1 million population)** | **COVID-19 case fatality ratio (number of deaths per 100 cases)** |
| --- | --- | --- | --- | --- | --- | --- |
| Andhra Pradesh | 13098 | 169 | 5908 | 242.99 | 3.14 | 2.78 |
| Assam | 7493 | 11 | 5089 | 210.44 | 0.31 | 0.22 |
| Bihar | 9224 | 62 | 7156 | 73.91 | 0.50 | 0.86 |
| Chhatisgarh | 2694 | 13 | 2062 | 91.52 | 0.44 | 0.63 |
| Delhi | 83077 | 2623 | 52607 | 4440.03 | 140.19 | 4.75 |
| Goa | 1198 | 3 | 478 | 755.24 | 1.89 | 0.62 |
| Gujarat | 31397 | 1809 | 22808 | 491.56 | 28.32 | 7.35 |
| Himachal Pradesh | 916 | 8 | 518 | 122.92 | 1.07 | 1.52 |
| Haryana | 13829 | 223 | 8917 | 490.31 | 7.91 | 2.44 |
| Jharkand | 2364 | 12 | 1793 | 61.25 | 0.31 | 0.66 |
| Jammu and Kashmir | 7093 | 94 | 4316 | 521.30 | 6.91 | 2.13 |
| Karnataka | 13190 | 207 | 7509 | 195.23 | 3.06 | 2.68 |
| Kerala | 4190 | 23 | 2150 | 117.37 | 0.64 | 1.06 |
| Madhya Pradesh | 13186 | 557 | 10084 | 154.48 | 6.53 | 5.23 |
| Maharashtra | 164626 | 7429 | 86575 | 1336.86 | 60.33 | 7.90 |
| Odisha | 6614 | 28 | 4743 | 142.68 | 0.60 | 0.59 |
| Punjab | 5216 | 133 | 3526 | 173.05 | 4.41 | 3.63 |
| Rajasthan | 17271 | 399 | 13611 | 213.14 | 4.92 | 2.85 |
| Tamil Nadu | 82275 | 1079 | 45537 | 1056.96 | 13.86 | 2.31 |
| Telangana | 14419 | 247 | 5172 | 366.31 | 6.27 | 4.56 |
| Tripura | 1351 | 1 | 1086 | 324 | 0.24 | 0.09 |
| Uttar Pradesh | 22147 | 660 | 14808 | 93.10 | 2.77 | 4.27 |
| Uttarakhand | 2823 | 38 | 2015 | 250.91 | 3.38 | 1.85 |
| West Bengal | 17283 | 639 | 11193 | 173.51 | 6.42 | 5.40 |
